# What could improve surgical data system at health facilities with high surgical volume in Ethiopia?

**DOI:** 10.1186/s12913-024-11303-6

**Published:** 2024-07-26

**Authors:** Melaku Godebo, Dawit Bete, Seye Minass, Tewodros Liyew, Ftalew Gebreyesus, Emily Bryce, John Varallo, Tigistu Ashengo

**Affiliations:** 1 Jhpiego, Addis Ababa, Ethiopia; 2 Jhpiego, Baltimore, United States; 3 Federal Ministry of Heath, Addis Ababa, Ethiopia

**Keywords:** Surgical data management, Data visualization, Hub-and-spoke mentorship, Performance review meetings

## Abstract

**Background:**

The effective management of surgical and anesthesia care relies on quality data and its readily availability for both patient-centered decision-making and facility-level improvement efforts. Recognizing this critical need, the Strengthening Systems for Improved Surgical Outcomes (SSISO) project addressed surgical care data management and information use practices across 23 health facilities from October 2019 to September 2022. This study aimed to evaluate the effectiveness of SSISO interventions in enhancing practices related to surgical data capture, reporting, analysis, and visualization.

**Methods:**

This study employed a mixed method, pre- post intervention evaluation design to assess changes in data management and utilization practices at intervention facilities. The intervention packages included capacity building trainings, monthly mentorship visits facilitated by a hub-and-spoke approach, provision of data capture tools, and reinforcement of performance review teams. Data collection occurred at baseline (February – April 2020) and endline (April – June 2022). The evaluation focused on the availability and appropriate use of data capture tools, as well as changes in performance review practices. Appropriate use of registers was defined as filling all the necessary data onto the registers, and this was verified by completeness of selected key data elements in the registers.

**Results:**

The proportion of health facilities with Operation Room (OR) scheduling, referral, and surgical site infection registers significantly increased by 34.8%, 56.5% and 87%, respectively, at project endline compared to baseline. Availability of OR and Anesthesia registers remained high throughout the project, at 91.3% and 95.6%, respectively. Furthermore, the appropriate use of these registers improved, with statistically significant increases observed for OR scheduling registers (34.8% increase). Increases were also noted for OR register (9.5% increase) and anesthesia register (4.5% increase), although not statistically significant. Assessing the prior three months reports, the report submissions to the Ministry of Health/Regional Health Bureau (MOH/RHB) rose from 85 to 100%, reflecting complete reporting at endline period. Additionally, the proportion of surgical teams analyzing and displaying data for informed decision-making significantly increased from 30.4% at baseline to 60.8% at endline period.

**Conclusion:**

The implemented interventions positively impacted surgical data management and utilization practice at intervention facilities. These positive changes were likely attributable to capacity building trainings and regular mentorship visits via hub-and-spoke approach. Hence, we recommend further investigation into the effectiveness of similar intervention packages in improving surgical data management, data analysis and visualization practices in low- and middle-income country settings.

## Introduction

Routine Health Information System (RHIS) is fundamental for low and middle-income countries (LMICs) to improve health care access, quality, disease surveillance and resource allocation [1–4].

Reliable RHIS data empowers countries to assess the health service performance towards universal health coverage targets and Sustainable Development Goals. However, despite its importance, RHIS implementation and its capability to generate reliable data to support evidence-informed decisions are often compromised in LMICs settings due to various factors [5–7]. These factors include political instability, corruption, weak governance, inadequate legal and regulatory frameworks, high trained staff turnover and limited training opportunities [8, 9].

Ethiopia’s RHIS has similarly struggled to generate quality data and foster evidence-based decision making to inform health programs. Technical and administrative limitations, such as insufficient training, resource shortage, inadequate supervision and weak governance, have been identified as key contributors [10, 11]. Recognizing this and for better value for money, the Ethiopian government has prioritized strengthening the health information system since the installment of the reformed health management information system (HMIS) in 2008. Thereafter, more resources were devoted to the information system during the first Health Sector Transformation Plan (HSTP-I 2016–2020) period; again, improving the health information system still was given priority in the second Health Sector Transformation plan (HSTP-II 2021-2025). Consequently, huge improvements have been achieved in health data management, health information system infrastructure, creating stewardship at all levels and assuring the basic principles of the health management information system reform that include standardization, integration, simplification and institutionalization [10, 12, 13]. However, challenges persist in data access, quality and utilization, necessitating further improvements in digitalization for optimal data storage, analysis, evidence generation, easy communication and evidence-based decision-making [12].

The Ethiopian Ministry of Health’s focus on the surgical and anesthesia care was evident in the first national Saving Lives Through Surgery (SaLTS-I) strategy (2016–2020). While SaLTS-I established surgical key performance indicators and integrated surgical data into the national health management information system, limitations persisted in data quality and use for informed decision-making [14].

To contribute to the improvement of surgical data management and information use, Jhpiego jointly with Government of Ethiopia implemented the System Strengthening for Improved Surgical Outcomes (SSISO) project across 23 health facilities (6 tertiary hospitals, 4 secondary hospitals, 5 primary hospitals and 8 health centers) in three regions from October 2019 to September 2022. This project primarily focused on improving surgical data management and evidence-based decision-making practices. The project’s baseline assessment revealed suboptimal surgical data management and evidence-based decision-making practice at intervention sites (unpublished data, 2020). Subsequently, the project implemented intervention packages which included capacity building trainings, monthly mentorship visits via hub-and-spoke approach, provision of data capturing tools and reinforcement of facility performance review teams.

Throughout the project period, a multifaceted approach targeting data system strengthening was implemented. This approach included capacity building trainings on surgical data management and information utilization for key personnel at each hub facility. These personnel included the HMIS focal person, quality improvement focal person and surgical unit coordinator. Following the training, these newly equipped experts provided monthly data mentorship to their own facility’s surgical team as well as 2–3 spoke facilities, employing a hub-and-spoke approach. Additionally, data capture tools such as standard surgical site infection logbooks, developed collaboratively with the MOH, were distributed to all intervention facilities. Furthermore, technical assistances on data quality and evidence-based decision-making was provided to facility performance review teams during quarter joint supportive supervisions and participatory review meetings.

This study aimed to evaluate effectiveness of these SSISO project interventions in enhancing surgical data management and evidence-based decision-making practice at the intervention sites.

## Methods

### Study design and setting

A pre- and post-intervention evaluation design using mixed method was undertaken across 23 health facilities in Ethiopia. These facilities served a combined catchment population of approximately 5 million in 2022. Facility selection was based on pre-defined selection criteria, including the availability of surgical care service, annual surgical volume and representation across all healthcare levels (tertiary, secondary, and primary). Geographically, the facilities were distributed across two regions (Amhara, Oromia) and one city administration (Addis Ababa). The intervention facilities encompassed a diverse range of facilities, including 6 tertiary level hospitals, 4 secondary level hospitals, and 13 primary care facilities.

To enhance knowledge transfer and capacity building, a unique hub-and-spoke approach was implemented. Hub facilities, typically tertiary or general hospitals with established expertise, resources, and prior mentoring experience, provided ongoing support on surgical care packages, including data management and evidence-based decision-making, to 2–3 spoke facilities. Spoke facilities, typically with less expertise, resources, and prior mentoring experience, were linked to their mentor hub facility within the existing referral system. This approach facilitated targeted intervention delivery and maximized the project’s reach within the healthcare network.

### Study subject identification and recruitment

Twenty-three HMIS focal persons from all project sites were surveyed to understand the data management and information use practice in their facility. For the qualitative method, purposive sampling strategy was employed to select key informants for in-depth interviews. These informants included individuals with crucial knowledge and experience in surgical services such as surgical service coordinators, surgeons, anesthetists/anesthesiologists, OR coordinators, and quality improvement focal persons. Selection prioritized maximum variation, considering different strata: hub facilities, spoke facilities and surgical service program lead units. Following this approach, 17 key informants in 4 facilities were selected from 33 potential participants at 8 hub facilities using maximum variation purposive sampling, and considering geographic location and level of facility mix. Similarly, 11 key informants in 4 spoke facilities were selected using same criteria, drawing from a pool of 45 potential participants at 15 spoke facilities. Finally, purposive sampling for maximum variation identified 4 key informants from regional health bureaus (RHB) and 2 from Ministry of Health (MOH), drawing from a pool of 6 potential participants at RHBs and 5 potential participants at MOH.

Surgical data documentation practice was evaluated by assessing source document availability and appropriate use for purposively selected registers. In each facility, the availability of the following registers was assessed: SSI logbook, referral register, OR register, anesthesia register, and OR scheduling register. The assessment further explored the appropriate use of the OR register, anesthesia register, and OR scheduling register.

Appropriate use of data capture tools was operationalized as the complete recording of selected key data elements within the registers for the past three months preceding the evaluation. (see box 1: operational definitions). Any missing data for the selected data elements within a register was considered indicative of inappropriate use for that specific register.

### Inclusion and exclusion criteria

Enrollment in and being supported by the SSISO project, and willing to participate in the study were considered as inclusion criteria. No exclusion criteria were applicable in this study.

### Data collection instrument

A semi-structured questionnaire was adapted from the routine data quality assessment tool of Federal Ministry of Health, national SaLTS initiative and performance monitoring plan of SSISO project. A data abstraction tool was adapted from the health data quality module of Federal Ministry of Health of Ethiopia [15]. Both tools were pre-tested at non-study sites for content, structure and response options.

A novel comprehensive in-depth interview guide was developed to gather detailed information on surgical service and anesthesia care including surgical data management, data visualization and evidence-based decision-making practice of surgical teams at intervention facilities, and the SaLTS team at regional health bureaus and the Ministry of Health. The interview guides were pre-tested at non-study sites for content, structure and clarity.

### Data collection procedure

Endline data collection was conducted from 4 to 15 July 2022 using pre-tested instruments: a semi-structured questionnaire, a data abstraction tool, and an interview guide. These instruments, with the exception of the interview guide, were also employed during the baseline assessment conducted from March 20, 2020 to June 20, 2020. The semi-structured questionnaire assessed surgical data management and information use practice within each intervention facility. The data abstraction tool facilitated the extraction of surgical data quality and key performance indicators from surgery program source documents.

To ensure effective communication, interview guides were translated into Amharic, the local language. All interviews were pre-arranged, conducted face-to-face at interviewee chosen venues, and facilitated by trained data collectors.

A total of 40 in-depth and key informant interviews (KII) were conducted to gather data on surgical services, anesthesia care, data management practices and evidence-based decision-making. These interviews focused on two groups of participants:

#### Key informants (*n* = 12)

This group comprised surgical service coordinators from MOH and RHB, as well as experts from professional associations. KIIs were interviewed to gain insight into institutional experiences and challenges related to the interventions.

#### In-depth interviews (*n* = 28)

This group included surgeons, anesthetists, OR coordinators, and quality improvement focal persons, representing both mentee and mentor facilities within the hub-and-spoke network. In-depth interviews explored individual experiences, encountered challenges, and overall recommendations regarding the interventions.

### Data quality assurance

To mitigate information and inter-observer bias among data collectors, several strategies were implemented. First, a two-day training was organized for data collectors and study supervisors. This training focused on the evaluation purpose, data collection tools, data quality principles, and ethical considerations. Additionally, operational definitions and standard operating procedures were developed and disseminated to ensure consistency in data collection practices.

Second, to minimize information bias, data collectors were recruited from project-supported health facilities but assigned to collect data at a different project-supported health facility. This approach reduced the likelihood of familiarity bias influencing their data collection process.

Third, data quality was further ensured through robust data verification procedures. Supervisors reviewed the collected data for consistency, accuracy and completeness before data collectors uploaded it to the online system. Jhpiego’s monitoring, evaluation and research team conducted additional data quality checks (consistency and completeness) by reviewing more than 50% of randomly selected data entries within the online system.

#### Consistency checks

Reviewed logically related data elements to identify potential inconsistencies.

#### Completeness checks

Compared the online data to corresponding hard copies to ensure all necessary information was captured.

### Data management and analysis

Data were collected using paper forms and subsequently entered into the CommCare mobile-based data collection system. The study team then exported the data from CommCare system to STATA v.17 for further analysis. Descriptive statistics were employed to characterize the prevailing status of metrics related to data management and information use practices. Data quality of selected data elements was determined by computing the verification factor (VF). This factor is defined as the ratio between the recounted number and the reported figure [15]. Recounted data originated from facility registers, while reported figures were obtained from DHIS2 system. The decision rule used for claiming data quality level were: VF ≤ 0.90 declared as over-reporting; 0.90 < VF < 1.10 declared as acceptable reporting; ≥ 1.10 declared as under-reporting. Paired sample proportions test with Z-statistic was used to determine statistically significant difference between baseline and endline values for availability and appropriate use of data capture tools, as well as performance review practices. Statistical significance was set at a *P*-value < 0.05.

The audio-recorded interviews were transcribed to English and aligned with the interviewers’ field notes. To ensure consistency, researchers reviewed transcripts while listening to the corresponding recordings. Thematic analysis was employed, with data analysis occurring with English scripts iteratively alongside data collection to facilitate saturation point identification, code development, and theme emergence. Themes were initially noted during transcription, with researchers revisiting the extracted transcripts to uncover deeper textual meaning. An inductive approach was used to develop the codebook transcript review. The codes were reduced and categorized based on logic, with descriptive data segments used to formulate themes. Thematic alignment with study objectives was ensured, and themes were presented with supporting verbatim quotes. ATLAS.ti© V.9 software was facilitated data reduction and analysis.

**Table:** 

Box 1. Operational definitions	
•**Availability of source document**: The presence of standard surgical data capturing tool of interest within the facility. •**Appropriate use of OR register**: Complete data availability for the following data elements within the past three months prior to the evaluation: Patient Identification, Procedure Type, and Operation Outcome. •**Appropriate use of Anesthesia register**: Complete data availability for the following data elements within the past three months prior to the evaluation: Patient Identification, Incision Time and Drugs used. •**Appropriate use of OR scheduling register**: Complete data availability for the following data elements within the past three months prior to the evaluation: Patient Identification, Perioperative Diagnosis and Date Surgery Scheduled. •**Spoke (mentee) facility**: A primary or secondary health facility with less expertise, resources, and prior mentoring experience, and linked to their hub facility within the existing referral system. •**Hub (mentor) facility**: A secondary or tertiary hospital with established expertise, resources, and prior mentoring experience, provided ongoing support on surgical care packages. •**Surgical data management** is the process of collecting, storing, organizing, protecting, and reporting data related to surgical procedures and outcomes. •**Evidence-based decision making** is the conscientious, explicit, and judicious use of facility generated evidence to make decisions about patient and facility management.	

## Results

This study was conducted in 23 health facilities: 6 tertiary hospitals, 4 secondary hospitals, 5 primary hospitals and 8 health centers. These facilities offered a total of 865 and 471 surgical beds in general and obstetric surgical wards, respectively. Notably, tertiary level hospitals held a disproportionate share of surgical bed capacity, accounting for 80% of beds in the general wards and 59% of beds in the obstetric wards (Table 1).

**Table 1:** Surgical system capacity at study sites, Apr – Jun 2022. (*n* = 23)

SN	Surgical System Capacity Metrics	Facility Type			
		Tertiary Hospital [*N* = 6]	Secondary Hospital [*N* = 4]	Primary Hospital [*N* = 5]	Health center [*N* = 8]
1	Average number of surgical beds in general surgical wards (mean, SD)	115.7 (63.3)	39.7 (14.2)	10.4(3.8)	NA
2	Average number of surgical beds in obstetric wards (mean, SD)	46.3 (32.4)	27.3 (9.2)	7.2 (3.4)	6 (2.1)
3	Average number of operation rooms (OR) for major obstetric and general surgeries (mean, SD)	9.7 (2.6)	5.5 (3.1)	1.8 (0 0.5)	1 (0)

At baseline, the availability of standard surgical data registers varied across the facilities. Only 26% of facilities had OR scheduling registers, 43.5% had referral registers, and none had SSI registers. By the project end period, the availability of these standard registers had increased significantly (*p*-value < 0.05) for all three types: OR scheduling (34.8% increase), referral (56.5% increase), and SSI (87% increase) (Table 2). Regarding the appropriate use of these registers, improvement were observed for OR registers (9.5% increase), Anesthesia registers (4.5% increase) and OR scheduling registers (34.8% increase). However, only the increase in appropriate use of OR scheduling registers was statistically significant [*p*-value < 0.05] (Table 2).

**Table 2:** Availability and appropriate use of standard registers at intervention sites during baseline and endline periods

SN	Data management Characteristics	Baseline (Feb- Apr 2020)	Endline (Apr – Jun 2022)	*P*-value
1	Availability of standard registers at facilities			
	OR register OR Scheduling register Anesthesia register Referral register SSI logbook (IPD) SSI logbook (OPD)	91.3% 26% 95.6% 43.5% 0% 0%	91.3% 60.8% 95.6% 100% 87% 69.6%	0.5 0.008 0.5 0.000 0.000 0.000
2	Facilities that use OR register appropriately	85.7%	95.2%	0.147
3	Facilities that use Anesthesia register appropriately	77.3%	81.8%	0.370
4	Facilities that use OR scheduling register appropriately	26%	60.8%	0.008

Endline data revealed a significant improvement in surgical service report submissions to the MOH/RHB. Submissions increased by 15% from the baseline value of 85%. Additionally, the proportion of surgical teams that analyzed and displayed data for informed decision-making rose from 30.4% at baseline to 60.8% at endline period [*p*-value < 0.05].

At endline, all facilities had source documents readily available for major surgical volume (MSV) and surgical site infection (SSI) indicators. Furthermore, 87.3% and 91% of facilities possessed source documents for surgical referral out (SRO) and institutional maternal mortality (IMM) indicators, respectively. Additionally, 21 (91.3%) health facilities reported cases that fall within the reporting period (Table 3). Such data was not captured during baseline and change in this parameter couldn’t be determined.

**Table 3:** The availability of source documents and reporting timeliness for selected indicators during endline period (Apr – Jun 2022)

Variables		Indicators for which source documents reviewed			
		Major Surgical Volume	Surgical Site Infection	Surgical Referral Out	Institutional Maternal Mortality
Health facilities that had source documents (n, %)		23 (100)	23 (100)	20 (87)	21 (91.3)
Health facilities that had complete source documents (n, %)		21 (91.3)	21 (91.3)	20(100)	21 (100)
Health facilities that reported cases that fall within the reported period (n, %)		23 (100)	22 (95.7)	20 (100)	21 (100)

The proportion of health facilities that had acceptable data quality for MSV, SSI, and IMM were 83% (19/23), 90% (17/19), and 100% (21/21), respectively (Table 4). Level of data quality on selected indicators was not captured during baseline and change in this parameter couldn’t be determined.

**Table 4:** Comparison of reported vs. recounted figures for selected indicators during endline period (Apr – Jun 2022)

Variables	# (%) of HFs over-reported	# (%) of HFs with an acceptable report	# (%) of HFs under-reported
Major Surgical Volume	2 (8.7%)	20 (86.9%)	1 (4.4%)
Surgical Site Infection	1 (5.3%)	17 (89.5%)	1 (5.3%)
Institutional Maternal Mortality	0 (0%)	21 (100%)	0 (0%)

At endline, the majority (82%) of health facilities had knowledge and experience sharing practices using different platforms such as morning sessions and workshops. Notably, health centers demonstrated that the strongest commitment to best practice documentation and colleague sharing, with 100% of facilities implementing this practice. Secondary and primary-level hospitals all had a mentoring system to support, guide and encourage new surgical staff. However, less than half of facilities have a debriefing session after the surgical procedure (Table 5).

**Table 5:** Learning and knowledge management practices at intervention sites during the endline period

Variables		Facility Type				Total *N* (%)
		Tertiary Hospital *N* (%)	Secondary Hospital *N* (%)	Primary Hospital *N* (%)	Health center *N* (%)	
Heath facilities that use virtual discussion forums to have quick discussions						
	Yes	5 (83.3)	4 (100)	1 (20)	2 (25)	12 (52.2)
	No	1 (16.7)	0	4 (80)	6 (75)	11 (47.8)
Health facilities that share knowledge or experience using platforms						
	Yes	5 (83.3)	4 (100)	2 (40)	8 (100)	19 (82.6)
	No	1 (16.7)	0	3 (60)	0	4 (16.4)
Health facilities that capture good practices and share with a work colleague						
	Yes	5 (83.3)	3 (66.7)	4 (80)	8 (100)	20 (87)
	No	1 (16.7)	1 (33.3)	1 (20)	0	3 (13)
Health facilities that have a mentoring system to support, direct and encourage new surgical staffs						
	Yes	5 (80)	4 (100)	5 (100)	7 (87.5)	21 (91.3)
	No	1 (20)	0	0	1 (12.5)	2 (8.7)
Heath facilities that have debriefing sessions after surgical procedures						
	Yes	4 (66.7)	2 (50)	2 (40)	3 (37.5)	11 (47.8)
	No	2 (33.3)	2 (50)	3 (60)	5 (62.5)	12(52.2)
Health facilities that discuss Surgical Safety Checklist data during debriefing sessions						
	Yes	4 (66.7)	1(25)	2 (40)	3 (37.5)	10 (43.5)
	No	2 (33.3)	3 (75)	3 (60)	5 (62.5)	13 (56.5)

### Qualitative study finding

A total of 40 individuals participated in the qualitative component of the study. Among these participants, 75% were men, and about 50% fell within the 31–40 year age range. Of the total, 15(37.5%) were specialists with different specialty in surgical care, and ten study participants were public health professionals. Eleven of them were operation room leaders, and nine were quality office heads.

Regarding organizational mix, the participants were from MOH, RHB, professional associations, Jhpiego, mentee and mentor facilities. Their professional roles encompassed health quality improvement, planning, and surgical service delivery. Importantly, the key informant group included individuals who were significantly involved during project implementation and possessed in-depth knowledge of surgical data management and evidence-based decision-making practices.

From the entire dataset, six themes were identified: hub and spoke mentorship model; documentation, data use and knowledge management; project impact; challenge during implementation; sustainability, scalability and partnership; and area of improvements (Table 6). One of the themes was related to surgical data management and evidence-based decision-making practice, and it is presented below with supportive verbatim quotes under subheadings: “Data Documentation”, “Surgical data use”, and “knowledge management practice”.

**Table 6:** Themes, categories and codes emerged from qualitative data analysis during the endline period

Themes	Categories	Codes
Hub and spoke mentorship model	Experience	Aim, plan, coordination, communication, schedule, context, incentive, benefit, challenge, gap, other model
	Insight	
Documentation, data use, and knowledge management	Documentation	Checklist, registry, forms, E- data, analysis, standard, feedback, meeting, use, dissemination
	Data quality	
	Data use	
Project impact	Benefit	Data use, Clinical skill, non-clinical skill, knowledge, coordination, support, team spirit, lessons
	Lessons learnt	
Challenge during implementation	Emergencies	Budget, commitment, motivation, withdrawal, transportation, incentive, burden, water, electricity, covid-19, security
	Resources	
	Workload	
Sustainability, scalability and partnership	Sustainability	Data quality, teamwork, mentor support, integration, experience sharing
	Scalability	
	Partnership	
Area of improvements	Teamwork	Incentives, Budget, commitment, motivation, withdrawal, transportation, incentive, burden, infrastructure
	Resources	
	Workload	
	Data quality and use	

#### Data documentation

Qualitative data revealed positive changes in surgical care documentation within patient charts and registration books. However, some participants from mentor and mentee facilities highlighted ongoing challenges, such as gaps in completing required data fields and delays in data sharing or reporting.

*“We report the mentorship data after one or two days of return with the link provided. In the beginning*,* we were working on surgical safety checklist utilization and then completeness. Currently*,* we are working on adherence*,* after its completeness is good.” (BSc*,* Nurse-OR head*,* mentor)*.

#### Surgical data use

Almost all study participants reported a shift in the way facilities utilized. Regular routine meetings were established among relevant healthcare professionals to discuss the data quality and identify areas for improvement. Data analysis informed decision-making related to service provision, including staffing plan, budget allocation, and infrastructural upgrade across all participating facilities. While participants acknowledged that data utilization was not yet fully optimized, a majority agreed that it had a clear positive impact on performance improvement within surgical and related services. The following verbatim quotes indicate how data was used.

*“We discuss our data in different meetings with ward nurses*,* OR team*,* and higher officials to put directions. Then the directions as a road map will be used by hospital staff to solve problems. There is little experience in using already collected data*,* but Jhpiego’s SSISO project insisted capacity building training and surgical team discussion*,* which has brought a change in data analysis and utilization.” (Surgeon-Surgery unit leader)*.

*“Barriers that are identified from the surgical safety checklist and surgical site infection data helped to make a decision. For example*,* in our last meeting*,* we discussed with the team not starting surgical operation before 2:30 am local time and then we decided to design an operation theater protocol (OTP).” (General Surgeon)*.

*“The data capture ability is improved than before. They are trained*,* and familiar with KPIs including the new staff recruited recently.” (BSc. Nurse-OR focal)*.

#### Knowledge management practice

Sharing hard copies, displaying data on dashboards, presenting findings during meetings, and utilizing mobile phone applications such as telegram were mentioned as a means of data presentation. Furthermore, some facilities reported posting data on websites and publishing finding in academic journals. These efforts to disseminate data and project experiences through scientific channels were identified as having significant benefits for communicating project activities and fostering engagement among healthcare professionals.

*“Recently we published two journals on biomedical central BMC journals. Above this*,* we also published different success stories on our organization website. The main purpose of all these things is*,* one same motivation factor especially those over worked health professionals*,* nurses*,* physicians*,* surgeons*,* Obstetricians*,* as you know the major thing for their motivation is recognition for their works”. (Surgeon-* medical chief officer*)*

## Discussion

This evaluative study demonstrated a significant increase in the availability of essential surgical data capture tools (OR scheduling register, Anesthesia register and SSI register) at the endline period compared to the baseline. This positive change was likely attributable to the project’s emphasis on mentorship. Monthly hub-and-spoke mentorship visits provided opportunities for project mentors to closely monitor the availability and use of standard registration books, offering guidance and feedback to address identified gaps. Recognizing the importance of data capture tools for generating comprehensive and accurate health data, a cornerstone of strong health system performance and decision-making [16, 17], the project, in collaboration with the Ministry of Health designed and introduced new surgical site infection registers for inpatient and outpatient departments which were not in place before the project. The Ethiopian health information system strategic plan (2020/21–2024/25) acknowledges the widespread complaint of recording tool shortages at regional and lower levels, highlighting the need to prioritize their availability [10]. The study also observed a statistically significant increase in the proportion of facilities submitting surgical reports to the ministry of health or regional health bureaus, rising from 85% at baseline to 100% at endline. This improvement in reporting was attributed to capacity building trainings and regular mentorship visits provided through SSISO project. The positive influence of mentorship and coaching on strengthening data management and quality were also reported in other studies conducted within Sub-Saharan Africa [18, 19].

Our study also showed that significant improvement in the appropriate use of OR scheduling register was observed compared to the baseline performance, and we trust the continuous and regular hub-and-spoke mentorship visits contributed to the improvement. This positive impact of mentorship on data management practices aligns with findings from other studies [18, 19]. The qualitative data also showed positive change in documentation practices at the study sites; but it was also pointed out that some important data, such as surgical site infection status, remained uncaptured within patient charts and registers. This may be linked to the negative attitudes among some healthcare providers who view data capture as a time-consuming or unnecessary activity, which was reflected during project performance meetings and site visits. Previous studies conducted in Ethiopia also showed that healthcare providers had unfavorable perceptions/attitude about and commitment to properly recording, reporting, and using health data [20, 21]. Likewise, no significant improvement was observed in appropriate use of available OR and anesthesia registers.

The study identified relatively low availability of source documents for SRO and IMM indicators. However, facilities possessing these source documents demonstrated good practices in maintaining completeness and reporting cases within the defined reporting period. On the other hand, though source documents for MSV and SSI were available in all project intervention sites, deficiencies were observed in completeness of source documents and reporting cases in their proper reporting period. These deficiencies in source documents and proper reporting was also observed in other health programs [22]. Additionally, MSV data tend to be more over-reported (greater than 10%) compared to SRO, SSI and IMM. A study conducted in 2016 in Ethiopia also revealed that the data system shortcomings in health service and program reporting, noting discrepancies between reported data and source documents [23]. Baseline data for these specific data quality metrics was unavailable, precluding comparisons of change over time.

The proportion of surgical teams that analyzed and displayed data during the endline period showed significant improvement compared to baseline values. Evidence from qualitative study also revealed a positive shift in data use culture at project intervention sites. These improvements are likely attributable to the capacity building trainings delivered at various intervals, as well as the regular hub-and-spoke mentorship visits, which consistently emphasized data use as a focus area. The positive contribution of training and mentorship in data analysis and visualization was also evidenced in other studies [18, 19]. However, limitations in data analysis and visualization practices persist, mirroring challenges faced by other low-and middle-income countries [7]. Another study in Ethiopia acknowledged improvements in data quality and use, but emphasized the need for further efforts to cultivate culture of information utilization within health facilities [21] .

A key strength of this study lies in its utilization of both quantitative and qualitative methods to gain a comprehensive understanding of data management and use practices within the project intervention sites. To our knowledge, this is the first study in Ethiopia to assess surgical data system in terms of data capture, reporting, analysis and visualization practices. In addition, the pre-post project evaluation design provides valuable evidence for shaping interventions in similar projects. The study limitation was the inability to determine changes in surgical data quality and evidence-based decision-making practices, as baseline data on these technical aspects was not collected.

## Conclusions

This study demonstrates the effectiveness of project interventions in improving surgical data management practices at project intervention sites. Compared to the baseline performance, significant improvements were observed in surgical data capture and reporting practice by the end of project period. These positive changes could likely be attributed to the project’s multifaceted approach, which included multiple rounds of capacity building trainings and hub-and-spoke mentorship visits.

In addition, the project’s emphasis on data utilization skills, including data processing, analysis, interpretation, and problem-solving, likely contributed to enhanced data analysis and visualization practices at the intervention sites. Equipping healthcare professionals with these knowledge and skills strengthens their ability to utilize data for informed decision-making.

To further strengthen surgical data management, data quality and evidence-based decision-making practice, future investments can prioritize interventions that address health care provider attitudes and perceptions regarding data quality and its value within their daily work routines. In addition, we recommend further investigation into the effectiveness of similar intervention packages in improving surgical data management, data analysis and visualization practices in low- and middle-income country settings.

## Data Availability

The data that support the findings of this study are available from Jhpiego Ethiopia but restrictions apply to the availability of these data, which were used under license for the current study, and so are not publicly available. Data are however available from the authors upon reasonable request and with permission of Jhpiego.
